# A rare case of concomitant Lambert–Eaton myasthenic syndrome and syndrome of inappropriate antidiuretic hormone secretion in a patient with small cell lung carcinoma

**DOI:** 10.1016/j.rmcr.2023.101930

**Published:** 2023-10-19

**Authors:** Yasuharu Oda, Hironobu Tsubouchi, Nobuyuki Ishii, Aya Kitamura, Eiji Moriyama, Eriko Mitsutome, Katsuya Sakai, Kazutaka Shiomi, Shigehisa Yanagi, Taiga Miyazaki

**Affiliations:** a Division of Respirology, Rheumatology, Infectious Diseases, and Neurology, Department of Internal Medicine, Faculty of Medicine, University of Miyazaki, Miyazaki, Miyazaki, Japan; b Chiyoda Hospital, Hyuga, Miyazaki, Japan; c National Hospital Organization Miyazaki Higashi Hospital, Miyazaki, Miyazaki, Japan

**Keywords:** Small cell lung carcinoma (SCLC), Lambert–Eaton myasthenic syndrome (LEMS), Syndrome of inappropriate antidiuretic hormone secretion (SIADH)

## Abstract

Small cell lung carcinoma (SCLC) is a neuroendocrine carcinoma with a poor prognosis and is a common cause of paraneoplastic syndromes. Paraneoplastic syndromes are characterized by neurological and endocrinological problems in patients with malignancy and are often associated with difficulty in induction of chemotherapy. Here we report the case of a patient with SCLC concomitant with two paraneoplastic syndromes, syndrome of inappropriate antidiuretic hormone secretion (SIADH) and Lambert–Eaton myasthenic syndrome (LEMS), who was treated with a platinum-doublet chemotherapy regimen. A 66-year-old male patient presented with a 1-month history of progressive proximal muscle weakness, ataxia gait and 5 kg of body weight loss. The laboratory tests revealed hyponatremia due to SIADH and the existence of antibodies against P/Q-type voltage-gated calcium channels. The nerve conduction study showed a low amplitude of compound muscle action potential (0.38 mv), a 34% decrement on 3-Hz stimulation, and a 1939% increment after maximum voluntary contraction in 10 seconds (7.75 mv). The endobronchial ultrasound transbronchial needle aspiration biopsy revealed the pathological findings of SCLC. A 2-cycle chemotherapy regimen of irinotecan plus cisplatin resulted in temporary tumor shrinkage that lasted 2 months, but the improvement of proximal muscle weakness and hyponatremia were maintained over the tumor re-progression period after chemotherapy. Although paraneoplastic syndromes accelerate the decrease in performance status, chemotherapy for SCLC may improve symptoms related to paraneoplastic syndromes and could be considered in similar cases.

## 1 Introduction

Lambert–Eaton myasthenic syndrome (LEMS) is caused by an autoimmune reaction against presynaptic P/Q-type voltage-gated calcium channels (VGCCs) that impairs the release of acetylcholine [1]. The clinical manifestations of the disease are late onset muscle fatigue and weakness, weight loss, and autonomic symptoms such as dry mouth and constipation [2,3]. LEMS may occur as an autoimmune disease without malignancy (NT-LEMS), or as a paraneoplastic disorder (CA-LEMS) that is most commonly associated with small cell lung carcinoma (SCLC) [2,3]. LEMS is a rare disease with a prevalence of 2.7 per million population, and 54%–71.4% of LEMS patients have concomitant or subsequent SCLC [3,4].

Syndrome of inappropriate anti-diuretic hormone secretion (SIADH) is characterized by dysregulated release of anti-diuretic hormone and subsequent retention of free water and is caused by infectious diseases, nervous system disorders, drug use and malignant tumors [5]. SIADH is the most important cause of hyponatremia in oncological and hospitalized patients. The incidence of SIADH in patients with SCLC is 7%–16% [6]. Since hyponatremia is a well-known prognostic and predictive factor in cancer patients and it has a negative influence on performance status and hospitalization [7], it is important to detect and treat paraneoplastic SIADH.

Here, we report a patient with LEMS and SIADH caused by SCLC, who was treated with platinum-doublet chemotherapy regimen.

## 2 Clinical report

A 66-year-old Japanese man with progressive muscle weakness and fatigue was admitted to the University of Miyazaki Hospital in December 2017. He had experienced difficulty walking and anorexia with 5 kg weight loss for the 1 month prior to admission. He smoked currently (46 pack-years), and only drank alcohol occasionally. His medical history included twice early-stage stomach cancer 2 and 11 years prior, and in both cases the cancers were resected by endoscopic submucosal dissection. His father died of lung cancer.

On admission, he complained of constipation, dry mouth and difficulty maintaining standing. He was alert and oriented, and his vital signs were within normal limits. His body weight was 44.9 kg, and his body mass index was 18.0 kg/m^2^. Physical examination revealed antecollis due to weak retroflexion of the head. On his neurologic examination, he presented muscle weakness of bilateral proximal limb muscles. He did not have any sensory disturbances or abnormal tendon reflexes.

**Fig. 1:**
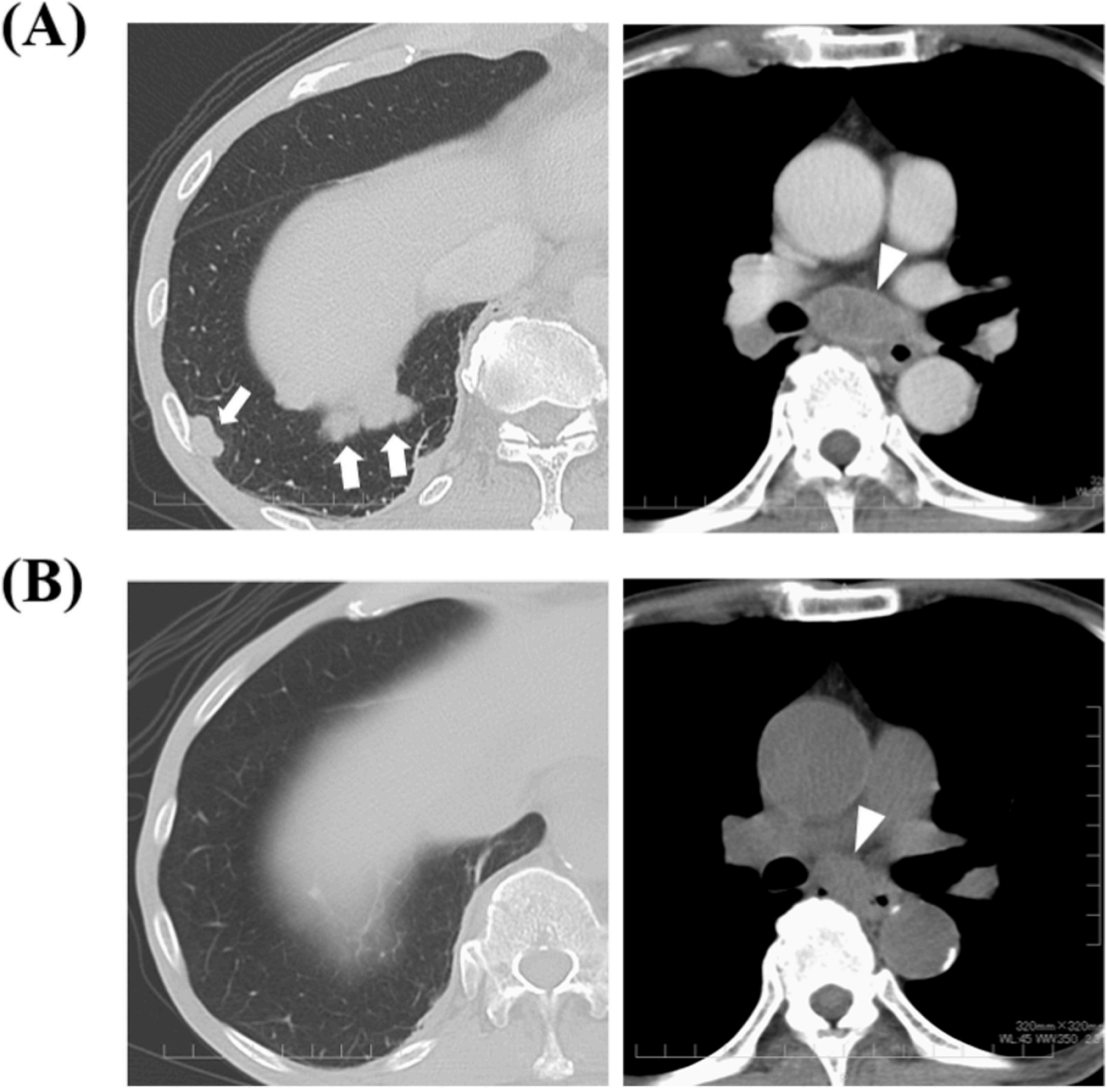
Chest CT at diagnosis (A) and 20 days after starting of chemotherapy (B). A: Chest CT at diagnosis showed multiple nodules in the right lower lobe (*arrows*) and subcarinal lymphadenopathy (*arrowhead*). B: Chest CT at 20 days after starting chemotherapy. Both the lung nodules and the lymph node metastasis were reduced in size (*arrowhead*).

His laboratory data were as follows: white blood cell count, 5,400/mm^3^; hemoglobin, 12.9 g/dL; platelet count, 136,000/mm^3^; total protein, 6.8 g/dL; blood urea nitrogen, 14.7 mg/dL; creatinine, 0.58 mg/dL; serum sodium, 130 mEq/L; serum potassium, 4.4 mEq/L; C-reactive protein, 0.14 mg/dL; pro-gastrin releasing peptide (Pro-GRP), 276 pg/mL; plasma osmolality, 264 mOsm/Kg; anti-P/Q-type voltage-gated calcium channel (VGCC) antibody, positive; anti-acetylcholine receptor, negative; antidiuretic hormone (ADH), 0.5 pg/ml; urine osmolality, 532 mOsm/Kg; and urinary sodium, 70 mmol/L. The laboratory data revealed that the patient's SCLC was complicated by SIADH. A chest computerized tomography (CT) scan revealed mediastinal and right hilar lymphadenopathy, and multiple nodules in the right subpleura (the maximum tumor diameter was 41 mm) [Fig. 1A]. No other organ showed signs of metastasis by chest and abdominal contrast-enhanced CT, head magnetic resonance imaging (MRI), or bone scintigraphy.

**Fig. 2:**
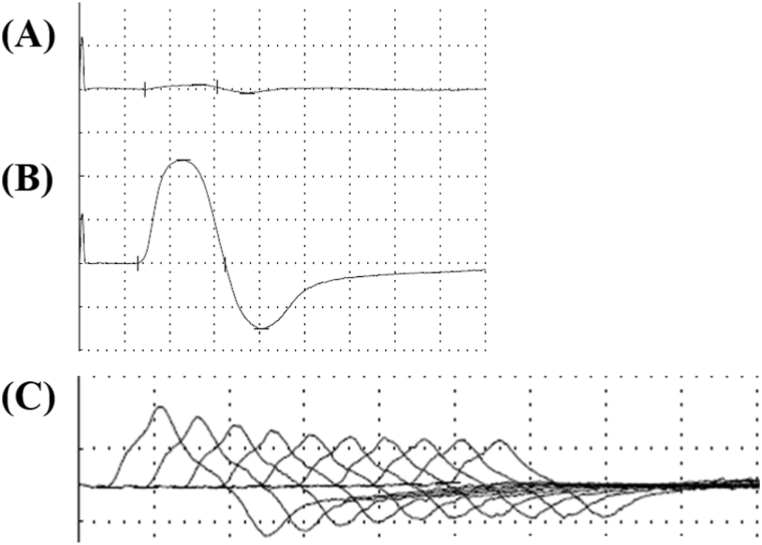
Repetitive nerve stimulation test. A: The compound muscle action potential (CMAP) by single‐pulse nerve stimulation of the left ulnar nerve in rest. The CMAP amplitude was 0.38 mV. B: The CMAP of single‐pulse nerve stimulation for the left ulnar nerve after 10 seconds of exercise. The CMAP amplitude was increase to 7.75 mV. C: The repetitive nerve stimulation test in the abductor digiti minimi muscle shows the waning phenomenon with 3-Hz repetitive stimulations of the left ulnar nerve. The 10th amplitude of the CMAP was decreased by 34% compared to that of the first CMAP.

In the nerve conduction study, the first compound muscle action potential (CMAP) amplitude by single‐pulse nerve stimulation during rest was small (0.38 mV), whereas the amplitude immediately after 10 seconds of exercise was markedly increased to 7.75 mV (Fig. 2A and B). In a 3 Hz repetitive nerve stimulation test of the left ulnar nerve, a 34% decrement was observed under the resting condition (Fig. 2C). LEMS was diagnosed as a result of these findings of the nerve conduction study and the positive result of anti-P/Q-type VGCC antibody.

The patient was diagnosed with SCLC, T4N2M0, stage 3B (UICC ver7), complicated by SIADH and LEMS. The specimen, collected by endobronchial ultrasound-guided transbronchial needle aspiration (EBUS-TBNA) to the subcarinal lymph nodes, included features of SCLC: rosette-like structures and cell clusters with a high proliferative ability and high nuclear-to-cytoplasmic ratio. Because lung metastatic lesions were spread into the right lung lobes, we provided platinum doublet chemotherapy.

**Fig. 3:**
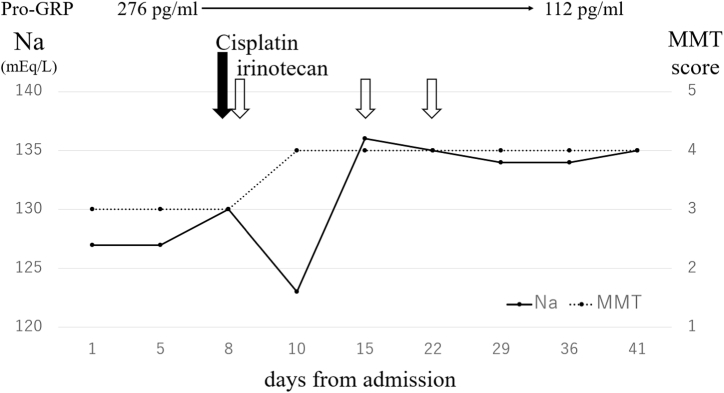
Changes in pro-gastrin releasing peptide (Pro-GRP) level, serum sodium level and manual muscle testing (MMT) score. Patient had chemotherapy on day 8 after admission. After cisplatin and irinotecan treatment, the Pro-GRP level, serum sodium level, and MMT score changed from 276 pg/ml to 112 pg/ml, from 127 mEq/L to 134 mEq/L, and from 3 to 4, respectively during the 28-days interval.

Systemic chemotherapy was initiated using a platinum-doublet chemotherapy regimen based on irinotecan (60 mg/m^2^, days 1, 8 and 15, intravenously) and cisplatin (60 mg/m^2^, day 1, intravenously) every 21 days as a first-line treatment immediately following diagnosis (Fig. 3). At 20 days after starting chemotherapy, a chest CT scan revealed a reduction of tumor size (reduction rate: 67%; Fig. 1B). The proximal muscle weakness gradually improved and allowed the patient to walk with a cane after chemotherapy. The manual muscle testing (MMT) score of lower extremity muscles was improved from 3 to 4 after 28 days of starting chemotherapy. As treatment for SIADH, fluids were restricted to 1000 ml/day during the hospitalization period. Although a fluid load of at least 1.5 L was administered to prevent renal damage due to cisplatin only for the first three days of chemotherapy, hyponatremia was confined to that period, and did not occur during the subsequent cycles of chemotherapy or additional lines of chemotherapy. Although tumor progression was observed after 2 cycles of first-line chemotherapy and the patient underwent second- and third-line chemotherapy with amrubicin and irinotecan, respectively, he died at 6 months after the SCLC diagnosis.

## 3 Discussion

Here, we report a case of paraneoplastic syndrome presenting with LEMS and SIADH in a patient with SCLC. Paraneoplastic syndromes, highly associated with SCLC, are clinical entities associated with cancers and often overlap with endocrine and neurological problems. Detection and management of paraneoplastic syndromes is thus an integral part of cancer management [8]. Although LEMS and SIADH are paraneoplastic syndromes, they can also be associated with benign disease [5,7,9]. Due to the complexity of symptoms in cases with paraneoplastic syndromes, there have been reports of delayed diagnosis of the underlying malignancy leading to worse prognosis [8]. Although it is difficult to completely eradicate all of the paraneoplastic syndrome-associated symptoms, the extent of the conditions is generally improved with successful treatment of the primary tumor.

In the present case, the levels of plasma osmolality, urine osmolality, urine sodium, serum sodium, serum creatinine and antidiuretic hormone, the non-use of diuretic agents, and the normal thyroid and adrenal function met the diagnostic criteria of SIADH [9], while the proximal muscle weakness, symptoms of autonomic disorder, such as dry mouth and constipation, higher anti-P/Q-type VGCC antibody, and features of nerve conduction studies—i.e., CMAP amplitude decrease, waning phenomenon by 3 Hz stimulation of the ulnar nerve and waxing phenomenon after 10 seconds of maximal muscle contraction—satisfied the diagnostic criteria of LEMS [2]. Cases with coexistent LEMS and SIADH are rare, and have mostly been described in the form of case reports [10–13, Table 1]. Among the 4 reported cases of SCLC with LEMS and SIADH [[10], [11], [12], [13]], the rates of symptom improvement in muscle weakness with LEMS, symptom improvement in hyponatremia with SIADH, and tumor shrinkage were 75%, 100% and 75% in patients receiving platinum-doublet chemotherapy, respectively. In addition, 3 of the 4 patients were alive at the time of the case report (4, 15, and 17 months), and one died at 4 months. In the present case, the chemotherapy resulted in temporary shrinkage of the SCLC lesions, but the patient died 6 months after diagnosis. Hyponatremia is an independently negative prognostic factor both for overall survival and progression-free survival, as are extensive stage, insufficient effect of first‐line chemotherapy and high neuron-specific enolase level [14]. Regarding the relationship between the prognosis of SCLC and LEMS, LEMS complications, unlike other paraneoplastic syndromes, have been reported to improve prognosis independently of prognostic variables such as lesion extent and performance status [15]. Since some reports indicate that complications with SIADH worsen the prognosis of patients with SCLC [7,16], the survival duration after diagnosis in the patients with LEMS and SIADH may be shorter than that in the usual SCLC with LEMS cases.

**Table 1:** List of previously reported SCLC patients of concomitant LEMS and SIADH.

Author	Kobayashi^10)^	Manji^11)^	Riva^12)^	Honoki^13)^	Present case
Age	61	67	57	68	66
Stage	ED	unknown	unknown	LD	ED
Treatment	CBDCA + etoposide	CPA + ADM + etoposide	CPA + VCR + etoposide + epiadriblastine	CBDCA + etoposide	CDDP + irinotecan
Effect to					
LEMS	improved	partially improved^a^	improved	improved	partially improved^a^
SIADH	improved	improved	improved	improved	Improved
**Tumor**	PR	PR	unknown	PR	PR
Survival duration (months)	≥17	≥15	4	≥4	6

Abbreviations: LD, Limited Disease; ED, Extensive Disease; CBDCA, Carboplatin; CPA, Cyclophosphamide; ADM, Adriamycin; VCR, Vincristine; CDDP, Cisplatin; PR, Partial Response.

a The patient partially got better, but not completely.

To assess the distribution of electrophysiological abnormality in the present case, we examined the abductor digiti minimi muscle (ADMM). Maddison et al. reported that despite predominantly proximal limb weakness seen in patients with LEMS, the most relevant muscles for detecting abnormalities in the evoked electromyography in patients with LEMS were ADMM, abductor pollicis brevis, and anconeus [17]. The evoked electromyography after chemotherapy was not performed in this case.

Although some treatment options are available for the symptomatic treatment of LEMS, such as amifampridine, guanidine, pyridostigmine, and intravenous immune globulin, chemotherapy for the primary tumor is the first priority in SCLC patients with LEMS [18,19]. Chalk et al. reported that 7 of 11 patients after chemotherapy exhibited substantial neurologic improvement [20]. In the present case, the proximal muscle weakness improved after the chemotherapy and the improvement was sustained until 4 months after the start of chemotherapy, when the patient's performance status worsened.

There was limitation to the differentiation of other neurological disease. The present case was diagnosed as LEMS based on the symptoms in addition to higher anti-P/Q-type VGCC antibody and abnormality of evoked electromyography. However, the needle electromyography, lumbar puncture, and screening test of autoantibodies associated with paraneoplastic neurological syndrome were not performed in the present case.

In summary, we have described the case of an SCLC patient with SIADH and LEMS who showed improvement of paraneoplastic symptoms, particularly proximal muscle weakness, after a platinum-doublet chemotherapy regimen. Although in SCLC cases with concomitant SIADH and LEMS, low PS due to muscle weakness and hyponatremia may affect the tolerability of chemotherapy, improvement of the paraneoplastic symptoms related to SIADH and LEMS may be achieved and maintained when the chemotherapy has been successful.

## Declaration of competing interest

The authors have no conflicts of interest to declare.

## References

[1] Lang B., Newsom-Davis J., Prior C., Wray D. (1983 Nov). Antibodies to motor nerve terminals: an electrophysiological study of a human myasthenic syndrome transferred to mouse. J. Physiol..

[2] Titulaer M.J., Lang B., Verschuuren J.J. (2011 Dec). Lambert-Eaton myasthenic syndrome: from clinical characteristics to therapeutic strategies. Lancet Neurol..

[3] Nakao Y.K., Motomura M., Fukudome T., Fukuda T., Shiraishi H., Yoshimura T., Tsujihata M., Eguchi K. (2002 Dec 10). Seronegative Lambert-Eaton myasthenic syndrome: study of 110 Japanese patients. Neurology.

[4] Yoshikawa H., Adachi Y., Nakamura Y., Kuriyama N., Murai H., Nomura Y., Sakai Y., Iwasa K., Furukawa Y., Kuwabara S., Matsui M. (2022 Sep 5). Nationwide survey of Lambert-Eaton myasthenic syndrome in Japan. BMJ Neurol Open.

[5] Mentrasti G., Scortichini L., Torniai M., Giampieri R., Morgese F., Rinaldi S., Berardi R. (2020 Jul 24). Syndrome of inappropriate antidiuretic hormone secretion (SIADH): optimal management. Therapeut. Clin. Risk Manag..

[6] Kanaji N., Watanabe N., Kita N., Bandoh S., Tadokoro A., Ishii T., Dobashi H., Matsunaga T. (2014 Aug 10). Paraneoplastic syndromes associated with lung cancer. World J. Clin. Oncol..

[7] Fiordoliva I., Meletani T., Baleani M.G., Rinaldi S., Savini A., Di Pietro Paolo M., Berardi R. (2017 Nov). Managing hyponatremia in lung cancer: latest evidence and clinical implications. Ther. Adv. Med. Oncol..

[8] Spiro S.G., Gould M.K., Colice G.L., American College of Chest Physicians (2007 Sep). Initial evaluation of the patient with lung cancer: symptoms, signs, laboratory tests, and paraneoplastic syndromes: ACCP evidenced-based clinical practice guidelines (2nd edition). Chest.

[9] Ellison D.H., Berl T. (2007 May 17). Clinical practice. The syndrome of inappropriate antidiuresis. N. Engl. J. Med..

[10] Kobayashi T., Watanabe T., Ashinuma H., Amano H., Kuroda F., Tada Y., Takiguchi Y., Hiroshima K., Tatsumi K. (2011 Mar). [A case of small-cell lung carcinoma accompanied by the syndrome of inappropriate secretion of antidiuretic hormone and Lambert-Eaton myasthenic syndrome]. Nihon Kokyuki Gakkai Zasshi.

[11] Manji H., Schwartz M.S., McKeran R.O. (1990 Aug). Lambert Eaton syndrome: autonomic neuropathy and inappropriate antidiuretic hormone secretion in a patient with small cell carcinoma of the lung. J. Neurol..

[12] Riva M., Brioschi A.M., Marazzi R., Donato M.F., Ferrante E. (1997 Jun). Immunological and endocrinological abnormalities in paraneoplastic disorders with involvement of the autonomic nervous system. Ital. J. Neurol. Sci..

[13] Honoki H., Kawagishi Y., Oda H., Miwa T., Inomata M., Fujita T., Matsui S., Kashii T., Maruyama M., Kobayashi M. (2003 May). [Marked improvement of Lambert-Eaton myasthenic syndrome resulting from treatment for small cell lung carcinoma]. Nihon Kokyuki Gakkai Zasshi.

[14] Liu C., Jin B., Liu Y., Juhua O., Bao B., Yang B., Liu X., Yu P., Luo Y., Wang S., Teng Z., Song N., Qu J., Zhao J., Chen Y., Qu X., Zhang L. (2023 Apr). Construction of the prognostic model for small-cell lung cancer based on inflammatory markers: a real-world study of 612 cases with eastern cooperative oncology group performance score 0-1. Cancer Med..

[15] Maddison P., Gozzard P., Grainge M.J., Lang B. (2017 Apr 4). Long-term survival in paraneoplastic Lambert-Eaton myasthenic syndrome. Neurology.

[16] Castillo J.J., Vincent M., Justice E. (2012). Diagnosis and management of hyponatremia in cancer patients. Oncol..

[17] Maddison P., Newsom-Davis J., Mills K.R. (1998 Aug). Distribution of electrophysiological abnormality in Lambert-Eaton myasthenic syndrome. J. Neurol. Neurosurg. Psychiatry.

[18] Mason W.P., Graus F., Lang B., Honnorat J., Delattre J.Y., Valldeoriola F., Antoine J.C., Rosenblum M.K., Rosenfeld M.R., Newsom-Davis J., Posner J.B., Dalmau J. (1997 Aug). Small-cell lung cancer, paraneoplastic cerebellar degeneration and the Lambert-Eaton myasthenic syndrome. Brain.

[19] Wang S., Bruzzi J., Rodriguez-Garza V.P., Komaki R.R. (2006 Jan). Lambert-eaton myasthenic syndrome in a patient with small-cell lung cancer. Clin. Lung Cancer.

[20] Chalk C.H., Murray N.M., Newsom-Davis J., O'Neill J.H., Spiro S.G. (1990 Oct). Response of the Lambert-Eaton myasthenic syndrome to treatment of associated small-cell lung carcinoma. Neurology.

